# Consistency of Regions of Interest as nodes of fMRI functional brain networks

**DOI:** 10.1162/NETN_a_00013

**Published:** 2017-10-01

**Authors:** Onerva Korhonen, Heini Saarimäki, Enrico Glerean, Mikko Sams, Jari Saramäki

**Affiliations:** Department of Neuroscience and Biomedical Engineering, School of Science, Aalto University, Espoo, Finland; Department of Computer Science, School of Science, Aalto University, Espoo, Finland

**Keywords:** Functional magnetic resonance imaging, Functional brain networks, Node definition, Region of Interest, Anatomical atlas, Brain parcellation

## Abstract

The functional network approach, where fMRI BOLD time series are mapped to networks depicting functional relationships between brain areas, has opened new insights into the function of the human brain. In this approach, the choice of network nodes is of crucial importance. One option is to consider fMRI voxels as nodes. This results in a large number of nodes, making network analysis and interpretation of results challenging. A common alternative is to use predefined clusters of anatomically close voxels, Regions of Interest (ROIs). This approach assumes that voxels within ROIs are functionally similar. Because these two approaches result in different network structures, it is crucial to understand what happens to network connectivity when moving from the voxel level to the ROI level. We show that the consistency of ROIs, defined as the mean Pearson correlation coefficient between the time series of their voxels, varies widely in resting-state experimental data. Therefore the assumption of similar voxel dynamics within each ROI does not generally hold. Further, the time series of low-consistency ROIs may be highly correlated, resulting in spurious links in ROI-level networks. Based on these results, we recommend that averaging BOLD signals over anatomically defined ROIs should be carefully considered.

## Author Summary

Network methods have opened new insights on structure and functional dynamics of the human brain. However, constructing functional brain networks is far from trivial—the neuroscientific community still lacks a standard definition of the nodes of brain networks. In the present article, we consider the two most commonly used approaches: using either imaging voxels or predefined Regions of Interest (ROIs) as nodes of the network. We investigate what happens when voxel-level signals are averaged for obtaining ROI-level networks. We introduce the concept of ROI consistency to characterize the similarity of the dynamics of voxels in an ROI. With the help of consistency, we show that although voxels in an ROI are assumed to behave similarly, this assumption does not hold for all ROIs.

In the functional brain network approach (Sporns, 2013a, 2013b; Wig, Schlaggar, & Petersen, 2011), the brain is depicted as a collection of nodes and links. Each node represents a brain area that is supposed to be functionally homogeneous, and links represent anatomical or functional connections between nodes. Studies of structural features of networks constructed from functional magnetic resonance imaging (fMRI) data have opened new insights on the dynamics and function of the brain (for reviews, see Bassett & Bullmore, 2009; Papo, Zanin, Pineda-Pardo, Boccaletti, & Buldú, 2014; Sporns, 2013b).

However, the reliability of fMRI network analysis and the factors that affect it have lately become a subject of discussion (Andellini, Cannatà, Gazzellini, Bernardi, & Napolitano, 2015; Aurich, Alves Filho, da Silva, & Franco, 2015; Braun et al., 2012; Shehzad et al., 2009; Shirer, Jiang, Price, Ng, & Greicius, 2015; Telesford et al., 2010). One of the critical questions is how to choose what the nodes represent. The anatomical locations and the number of nodes have been reported to affect properties of brain networks, such as small-worldness or scale-freeness (de Reus & Van den Heuvel, 2013; Sporns, 2013a, 2013b; Wang et al., 2009; Zalesky et al., 2010).

It would be natural to consider single neurons as network nodes, linked by synaptic connections. However, this micro-level approach is not an option for studying the whole human brain, because of the excessive number of neurons and the lack of resolution of imaging methods (de Reus & Van den Heuvel, 2013). Thus, network studies of the brain are currently limited to the mesoscopic and macroscopic levels. For fMRI, these are the level of imaging voxels and the level of Regions of Interest (ROIs) that are collections of voxels defined on the basis of, for example, anatomical landmarks.

In the voxel-level approach, the nodes are voxels, cubical volume elements with 2–8 mm edges that form a regular grid covering the brain. The BOLD signal associated with each voxel is directly given by the fMRI measurement (Stanley et al., 2013). In the ROI-level approach, the nodes are ROIs that comprise tens to hundreds of voxels. An ROI’s signal is typically computed by averaging the BOLD signals of its voxels (Stanley et al., 2013). ROIs are usually defined using an anatomical atlas, based on structural MR images or histological investigations (see, e.g., Stanley et al., 2013). Data-driven methods that cluster voxels based on resting-state functional connectivity (e.g., Craddock, James, Holtzheimer, Hu, & Mayberg, 2012; Nelson et al., 2010; Power et al., 2011; Shen, Tokoglu, Papademetris, & Constable, 2013; for a review see Sporns, 2013b; Wig, Schlaggar, & Petersen, 2011), ICA, and dual regression (Beckmann, Mackay, Filippini, & Smith, 2009), or combination of anatomical, functional, and connectivity data (Fan et al., 2016; Glasser et al., 2016) have also been suggested for defining ROIs. However, despite promising results, these methods are used only infrequently.

The main benefits of the ROI approach are increased signal-to-noise ratio (SNR) and decreased computational cost. Further, one can expect that cognitive functions cover brain areas larger than single voxels (Shen et al., 2013; Wig et al., 2011). Therefore, the ROI approach may characterize true brain activity better than voxels with functionally arbitrary boundaries. However, areas related to cognitive functions may not necessarily match with the anatomical boundaries that define ROIs: The same function may be distributed across multiple anatomical areas, or one anatomical area may contain several functionally distinct subareas (Papo, Zanin, & Buldú, 2014; Stanley et al., 2013). Thus, the main disadvantage of the ROI approach is the possible loss of information that results from averaging signals of voxels that represent different functions for producing the ROI signal (Stanley et al., 2013).

On the other hand, if the functional network is constructed with voxels as nodes, problems arise from the numbers of nodes and links that represent correlations between voxel time series. Because of computational limitations, one has to heavy-handedly threshold correlation matrices to reduce the number of links. This results in sparse networks where much information has been discarded.

In both approaches, some information is deliberately discarded. But do they retain *similar* information? There is some evidence that moving from the voxel level to the ROI level changes network properties. Hayasaka & Laurienti (2010) found that ROI-level networks are less robust against fragmentation than voxel-level networks at low network densities. Further, ROI-level networks showed less small-world properties, and had different degree distributions and less stable hubs. Tohka, He, and Evans (2012) reported similar results in structural brain networks that were based on the thickness of cortical gray matter. Therefore, one may ask whether voxel-level and ROI-level networks provide comparable views on the underlying brain function.

In this article, we ask what happens to network connectivity when moving from the voxel-level network to the ROI level, where in both cases links represent zero-lag correlations. In particular, we focus on whether voxels of an ROI display coherent dynamics, as they should if the ROIs match with underlying functional areas. To this end, we introduce the concept of ROI *consistency* that quantifies the similarity of the signals of voxels that comprise the ROI, defined as their mean Pearson correlation coefficient. With the help of resting-state fMRI data of 13 subjects measured in-house as well as 28 subjects from the Autism Brain Imaging Data Exchange (ABIDE) initiative, we show that consistency varies widely across ROIs. Therefore voxel dynamics within ROIs are not always coherent. We then show that this variation is reflected in network properties: Signals of voxels in non-consistent ROIs are not correlated between ROIs, and less consistent ROIs are less central as nodes of the functional brain network.

## RESULTS

### Voxel Time Series Within ROIs Are on Average Correlated, but Not Uniformly

We begin by considering the functional homogeneity of voxels within ROIs—in the ROI approach, it is assumed that voxels within an ROI are functionally more similar and thereby have more strongly correlated time series than voxels in different ROIs. For defining ROIs, we use three different atlases: the anatomical Harvard-Oxford (HO) and Automated Anatomical Labeling (AAL) atlases as well as the Brainnetome atlas that is based on structural and functional connectivity.

For testing the homogeneity assumption, we calculated the distribution of Pearson correlation coefficients between the time series of pairs of voxels that are in the same ROI. This distribution was calculated across all ROIs and therefore covered the whole cerebral cortex and subcortical structures. In HO and AAL, also cerebellum was included; the Brainnetome atlas does not contain cerebellar ROIs. As a reference, we computed a similar distribution for pairs of voxels that reside in different ROIs.

**Figure 1.**
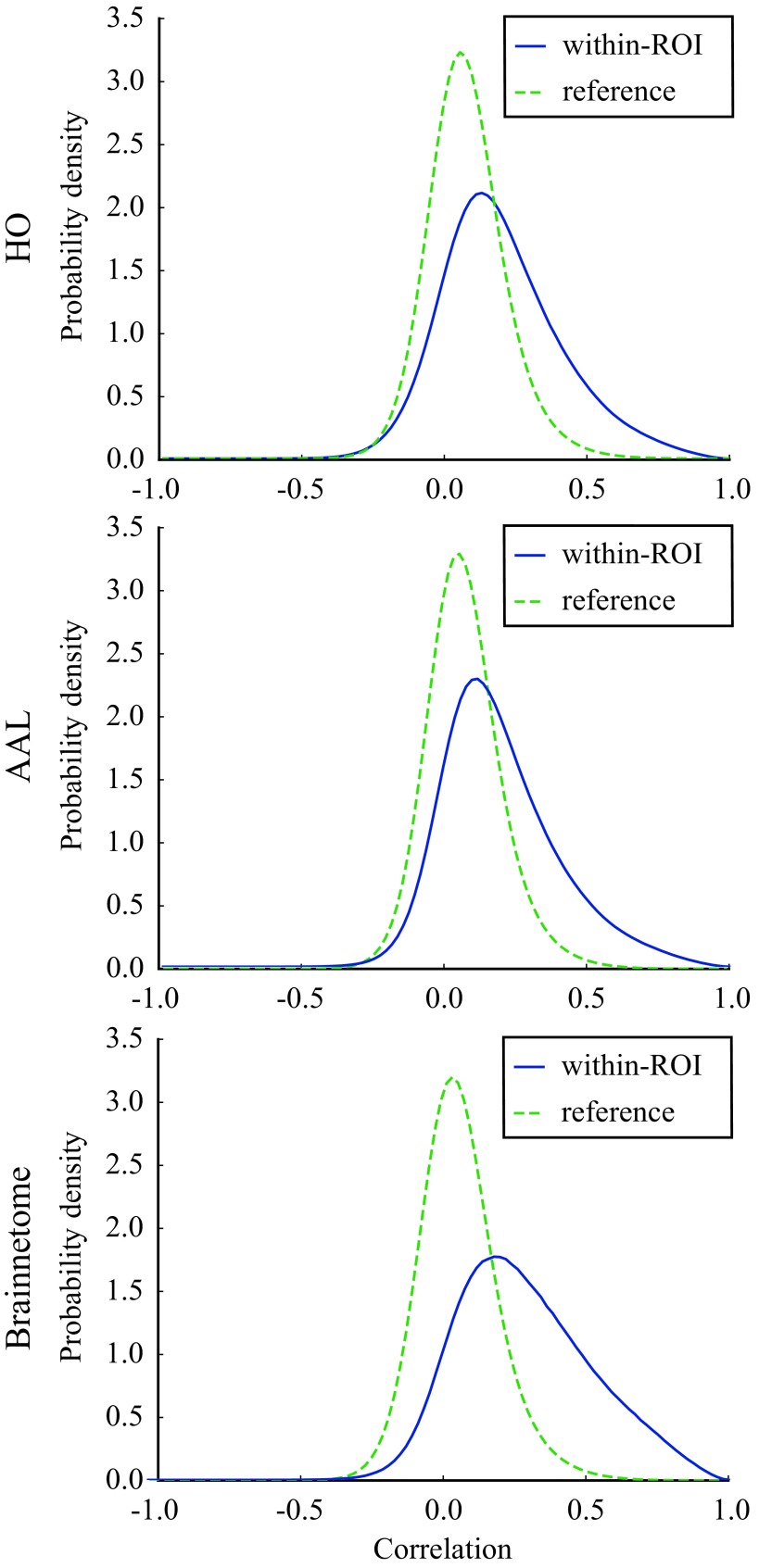
Voxel time series are on average more strongly correlated within ROIs than between ROIs but the correlation is not perfect. Distributions of Pearson correlation coefficients between voxel time series within (blue) and between (green, reference) ROIs in HO, AAL, and Brainnetome. Although the within-ROI correlation distribution is more skewed to the right, the distributions largely overlap, and small and negative correlations exist also within ROIs. Correlation distributions are calculated across all ROIs from the pooled data of 13 subjects.

Correlations are on average stronger within ROIs than in the reference (i.e., voxels in different ROIs) in all investigated parcellations: Their distribution has a higher mean (HO: *r* = 0.20 vs *r* = 0.073, Student’s *t* = 49.81, *p* ≪ 10^−5^; AAL: *r* = 0.19 vs. *r* = 0.067, *t* = 50.00, *p* ≪ 10^−5^; Brainnetome: *r* = 0.29 vs. *r* = 0.073, *t* = 83.05, *p* ≪ 10^−5^; the *p* value has been calculated using a permutation-based two-tailed *t* test; Glerean et al., 2016) and is more strongly right-skewed than the approximately normal reference distribution (Figure 1). However, there is a large overlap between the two distributions, and a significant number of small and negative correlations is present in both distributions. Thus, the functional uniformity of ROIs is far from perfect.

One might expect the connectivity-based Brainnetome parcellation to show stronger functional homogeneity of ROIs than the anatomical HO and AAL. Surprisingly, this is not the case: Although the mean of the within-ROI correlation distribution is sligthly higher in Brainnetome than in other parcellations, the overlap of the within-ROI and reference correlation distributions is equally large in all parcellations.

In order to investigate to what extent our results generalize to other datasets, we repeated all analysis of this article on a second, independet dataset, the ABIDE data (Di Martino et al., 2014). The results obtained using the ABIDE data were highly similar to those obtained using the in-house dataset and presented in this Results section. A detailed description of the results obtained using the ABIDE dataset can be found in Supplementary Results (Korhonen, Saarimäki, Glerean, Sams, & Saarimäki, 2017).

Spatial smoothing, a commonly used method in the preprocessing of fMRI data, makes the time series of neighboring voxels more similar, and is therefore expected to affect the results presented above. We repeated all analysis of this article for data that were smoothed with three Gaussian kernels with different full widths at half maximum. Although spatial smoothing increased all correlations between voxel time series, it has no qualitative effects on the results; for example, correlation distributions are as equally broad after spatial smoothing as without it (for a detailed description of the effects of spatial smoothing, see Supplementary Results; Korhonen et al., 2017).

### The Consistency of ROIs Varies Across the Brain

**Figure 2.**
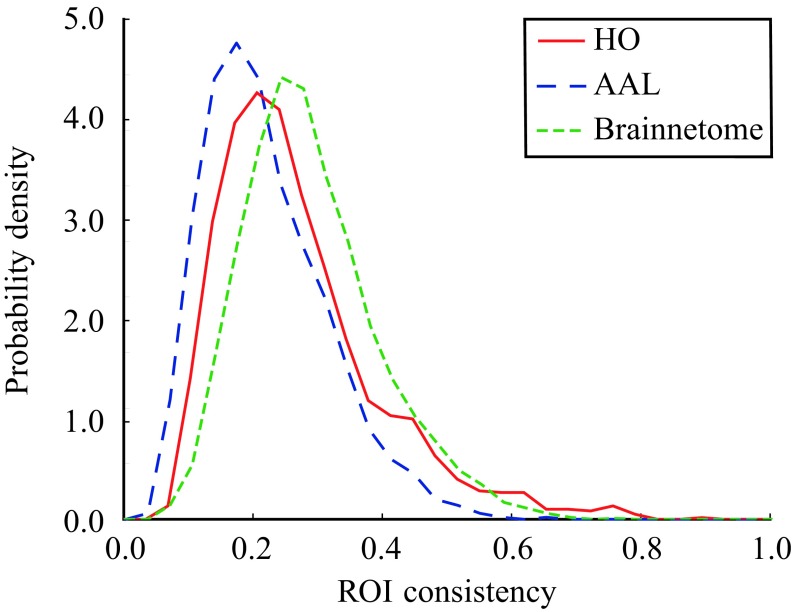
Consistency of ROIs is widely distributed. The consistency distributions has been calculated from the pooled data of 13 subjects.

The consistency of an ROI (*ϕ*), defined as the mean Pearson correlation coefficient of the time series of voxels belonging to the ROI, is a simple measure of the ROI’s functional cohesion. In order to evaluate the variation of consistency between ROIs, we calculated consistency distributions for each parcellation from the pooled data of all 13 subjects (Figure 2). Although the maximum observed ROI consistency is high (HO: consistency distribution is broad, peaking at low consistency values (HO: are not functionally uniform in the resting state.

**Figure 3.**
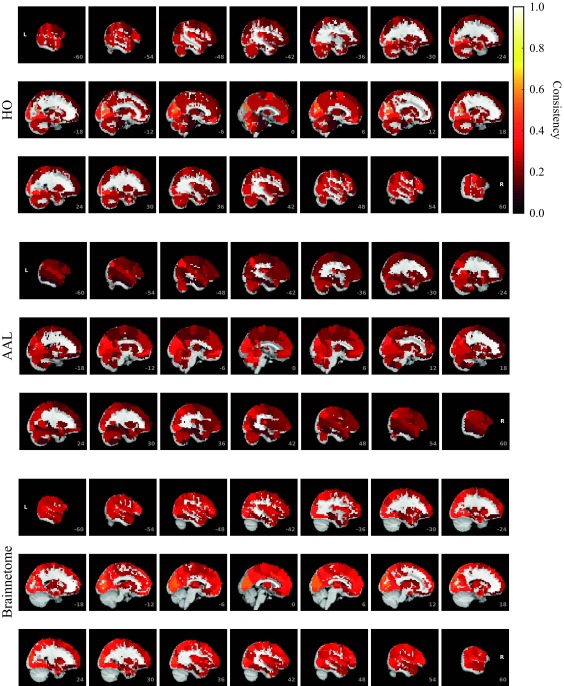
ROI consistency is not anatomically uniform. We have visualized the mean consistency across 13 subjects on a template brain. ROIs colored with yellow and orange are those with highest consistency, while low-consistency ROIs are colored with black. See the main text for the details of the identity of these ROIs. Areas colored with gray-white scale (white matter and, in the case of Brainnetome, cerebellum) are not included in this study.

To investigate in more detail how consistency is distributed among ROIs, we calculated the mean consistency for each ROI across subjects. Visualization of the mean consistency on a brain template (Figure 3) demonstrates what the broad consistency distribution means in practice: The most consistent ROIs have twice the consistency of the least consistent ones.

The identity of the most consistent ROIs varies across subjects only slightly. In HO, the most coherent ROIs include the left and right supracalcarine, cuneal, and intracalcarine cortices as well as the cerebellar areas Vermis Crus II and Vermis VIIb. In the case of the supracalcarine cortices and the cerebellar areas, the high consistency is probably explained by the size of the ROIs, since all these areas are small (containing 10 voxels or less).

In AAL, the most consistent ROIs include left and right cuneal cortex and right Heschl’s gyrus as well as the small cerebellar ROIs Vermis_1_2 and Vermis_10. In Brainnetome, the most consistent ROIs include several subsections of the left and right cuneal cortex and right medial superior occipital gyrus.

For the least consistent ROIs, intersubject variation is larger. In HO, the least consistent ROIS include the right frontal orbital cortex, right and left frontal pole, right precentral gyrus, and superior division of left lateral occipital cortex. In AAL, among the least consistent ROIs are left and right hippocampus, left and right inferior temporal gyrus, and left frontal superior orbital gyrus. In Brainnetome, the least consistent ROIs include left and right orbital gyrus (6_3), left and right fusiform gurys, and left and right hippocampus.

ROIs of different parcellations do not perfectly match with each other. However, it is interesting to see that same ROIs, in particular cuneal cortices, are among the most consistent ROIs in all three parcellations. For sizes, mean consistency values, and consistency ranks of all ROIs, see Supplementary Table (Korhonen et al., 2017).

In terms of mean consistency, the differences between the three parcellations investigated are small. Taking into account the individual variation of structure and function of the brain, one might expect to see more variation in consistency between subjects in Brainnetome than in HO and AAL. In order to test this hypothesis, we calculated the standard deviation of consistency across subjects for each ROI. However, we found no significant difference between the standard deviations of consistency in different parcellations.

**Figure 4.**
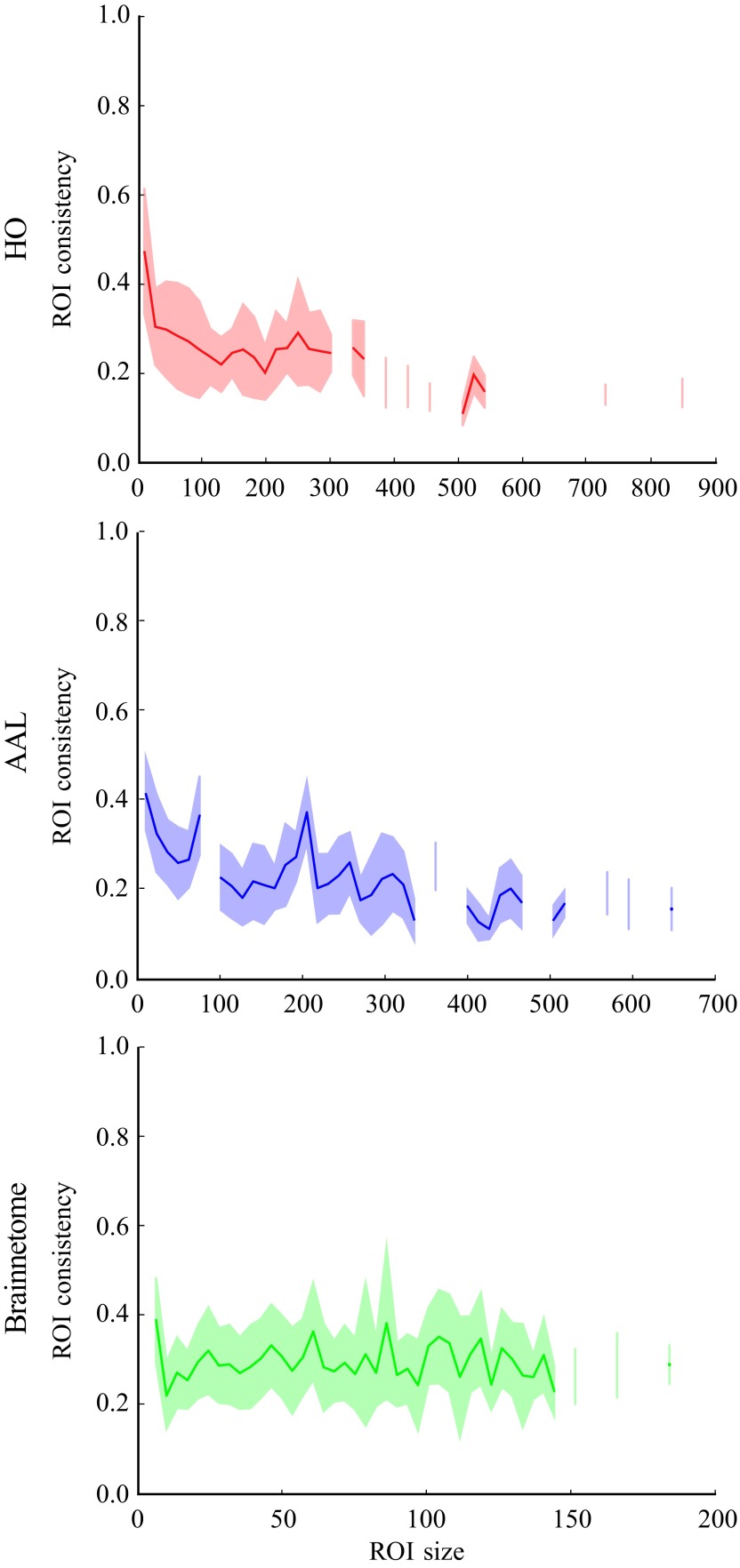
The size dependence of the average ROI consistency (line) and its variation (shadowed area) show that, in HO and AAL, small ROIs have on average higher consistency. This dependency is less visible for ROIs larger than 100 voxels. However, the variation of consistency is large for all ROI sizes. Therefore, the size of an ROI is not the only factor that affects its consistency. In Brainnetome, ROI size and consistency are not correlated. The consistency of each ROI has been pooled across 13 subjects, binned on the basis of ROI size, and then bin-averaged.

In HO and AAL parcellations, ROI consistency is partially explained by its size, that is, the number of voxels within the ROI (Figure 4). Consistency is highest for small ROIs and de creases with increasing ROI size (HO: Pearson correlation coefficient *r* = −0.35, *p* ≪ 10^−5^; AAL: *r* = −0.38,*p* ≪ 10^−5^). This decrease saturates for ROIs larger than a few hundred voxels. However, for ROIs of any size, there is a lot of variation around the mean. In Brainnetome, in contrast, ROI consistency and size do not correlate (*r* = 0.017, *p* = 0.35).

We also investigated whether head motion explains some part of the individual differences in ROI consistency. However, we did not find significant correlation between ROI consistency and subjects’ head motion, measured in terms of framewise displacement.

### Low ROI Consistency Predicts Low Voxel-Level Correlations Between Pairs of ROIs

One of the aims of this paper is to understand how the consistency of ROIs relates to their network properties. We begin by looking at the voxel level, and define the voxel-level correlation between two ROIs as the mean Pearson correlation coefficient between time series of voxels within the ROIs (see Equation 3). We expect that the voxel-level correlation is strong only if the ROIs are consistent enough. This is motivated as follows: A strong voxel-level correlation means that many pairs of voxels in both ROIs must have similar time series. This means that in each ROI, there must be a lot of similarly behaving voxels, resulting in high consistency. As the limiting case, ${\langle C\left(I,J\right)\rangle }_{vox}=1$ if and only if the time series of all voxels in both ROIs are equal (*x*_*i*_ = *x*_*j*_∀*i* ∈ *I*, *j* ∈ *J* and *x*_*i*_ = *x*_*i*′_ ∀*i*, *i*′ ∈ *I*). In order to test this hypothesis, we investigate voxel-level correlations between two ROIs as a function of the mean consistency of the ROI pair.

**Figure 5.**
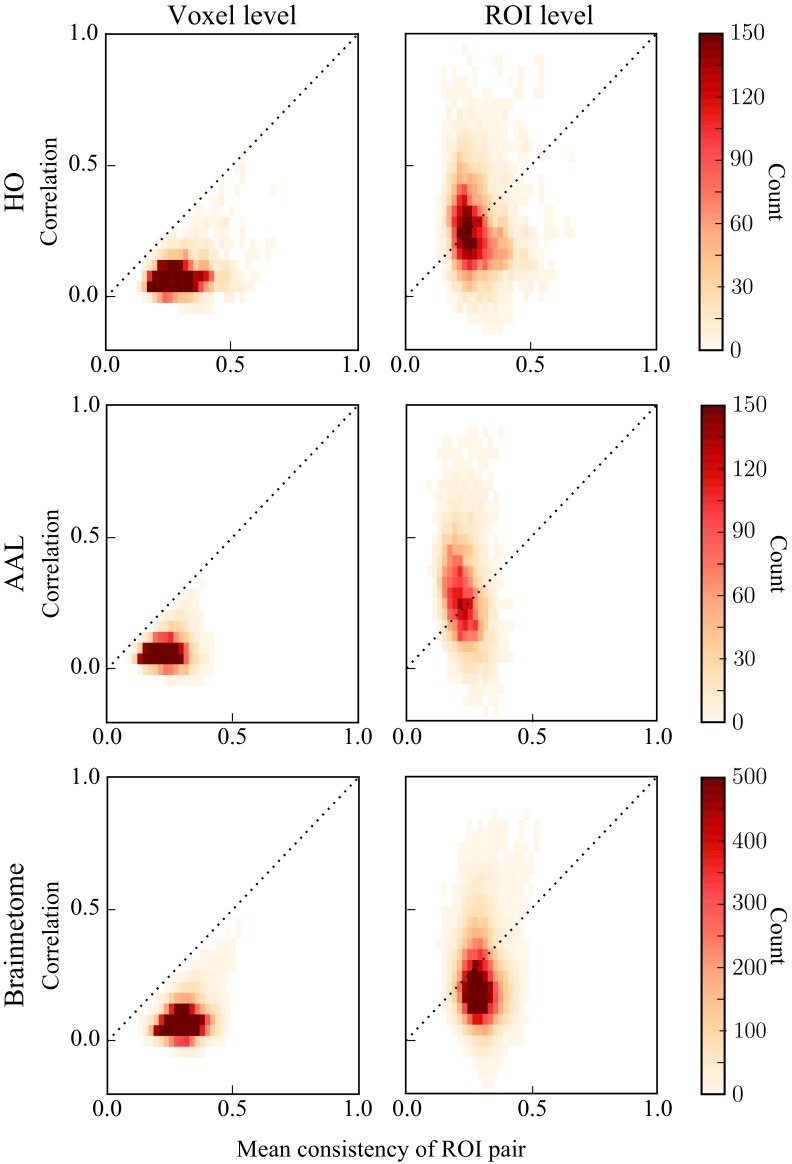
Averaging of voxel signals may induce spuriously strong ROI-level correlations between ROIs that have low consistency. Left: The relationship between voxel-level correlation and the mean ROI consistency for each ROI pair. Voxel-level correlation correlates with consistency, and all data points are located below the identity line. Right: The same relationship for ROI-level correlations and mean ROI consistency. ROI-level correlations are not correlated with consistency, and strong ROI-level correlations also exist between ROIs that have low consistency and weak voxel-level correlation. In order to produce the heatmaps, the consistency and voxel and ROI-level correlations have been averaged across 13 subjects.

As we hypothesized, our data indicate that high mean consistency is a prerequisite for a strong voxel-level correlation between two ROIs (Figure 5, left). However, it is clear that there is a lot of variation: Voxel-level correlation correlates only moderately with ROI consistency (HO: Pearson correlation *r* = 0.29, *p* ≪ 10^−5^; AAL: *r* = 0.17, *p* ≪ 10^−5^; Brainnetome: *r* = 0.28, *p* ≪ 10^−5^). Despite this variation, voxel-level correlations are never strong between low-consistency ROIs, and all data points are located below the identity line (${\langle C\left(I,J\right)\rangle }_{vox}=\phi$).

### Low-Consistency ROIs May Have High ROI-Level Correlations

Next, we investigated how ROI consistency relates to correlations at the ROI level. To this end, for pairs of ROIs, we calculated the ROI-level correlations (see Equation 4) as a function of their mean consistency. In HO and Brainnetome, ROI-level correlations depend on consistency clearly less than the voxel-level correlations (Figure 5, right; HO: Pearson correlation *r* = −0.14, *p* ≪ 10^−5^; Brainnetome: *r* = −0.00057, *p* = 0.92), whereas in AAL ROI consistency and ROI-level correlation are negatively correlated (*r* = −0.24, *p* ≪ 10^−5^).

As seen in Figure 5 (right), there are pairs of low-consistency ROIs that nevertheless display strong ROI-level correlations. From the functional point of view, this situation is not straightforward to interpret: If strong ROI-level correlations are taken as a sign of a strong functional relationship, how can this relationship be real if the ROIs themselves are nonuniform and lack consistency?

As one possible reason for these spurious-looking correlations, let us assume that signals of voxels in ROIs *I* and *J* share a common component, but their pairwise correlations are weak because of components unique to the signal of each voxel. In this case, the consistency of both ROIs is low. Then, the time series of the ROIs are obtained as averages over the time series of their voxels (see Equation 1). This averaging amplifies signal components shared by all voxels within the ROI and suppresses components unique to each voxel. Therefore, the time series of ROIs *I* and *J* consist mostly of the shared signal component and are strongly correlated.

The signal component shared by each voxel in the ROIs *I* and *J* may be either noise or true signal. Similarly, the suppressed signal components may be either true signal components unique to single voxels or independent noise. Based on the results visualized in Figure 5, it is not possible to say for sure whether the links between low-consistency ROIs are spurious or whether they are true correlations that have become visible when noisy signal components have been suppressed. However, this result suggests that extra care is needed when interpreting the ROI-level correlations, since any artifactual signal shared between voxels can induce spurious ROI-level correlations between ROIs that have low consistency.

**Figure 6.**
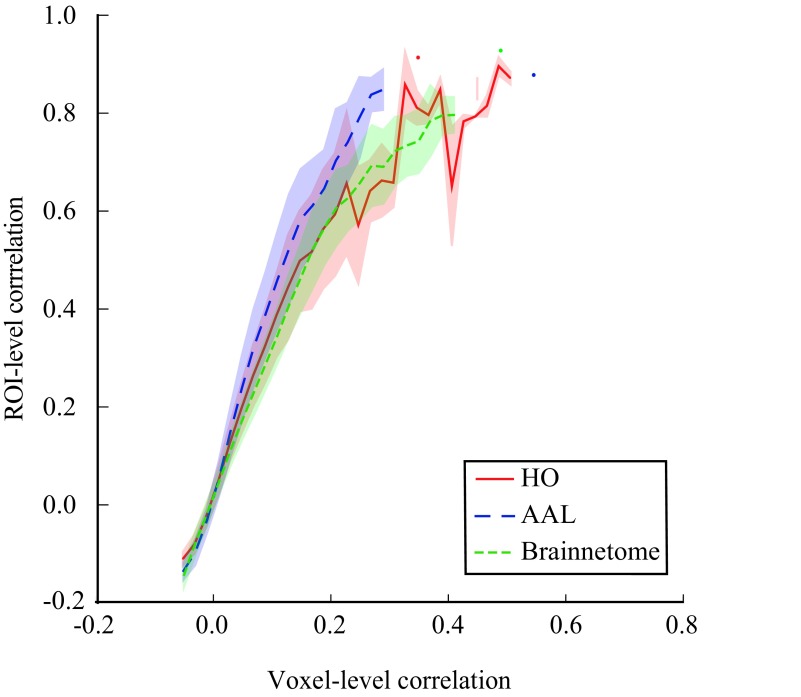
ROI-level correlations are stronger than the voxel-level correlations between the same ROIs. This is because averaging the voxel signals in order to obtain the ROI time series suppresses individual signal components of each voxel and amplifies components shared among voxels (for details and discussion, see the main text). Averaging and binning as in Figure 4.

For a concrete example of the effects of amplification and suppression of voxel-level signal components when moving to the ROI level, we investigated ROI-level correlations as a function of voxel-level correlations (Figure 6). We observed that in all investigated parcellations, on average ROI-level correlations increase faster than the increasing voxel-level correlations: Pairs of ROIs that correlate only moderately at the voxel level can appear highly correlated at the ROI level. For instance, an ROI pair that has a voxel-level correlation of *r* = 0.2 can have an ROI-level correlation as high as *r* = 0.6. This is, of course, a direct result of the averaging of voxel signals in order to obtain the ROI time series.

Further, the relationship between ROI-level and voxel-level correlations is nonlinear and saturates already at rather low voxel-level correlation values. Therefore, pairs of ROIs that appear to have similar correlations at the ROI level may in fact clearly differ at the voxel level.

### Consistency of an ROI Predicts Its Network Properties

**Figure 7.**
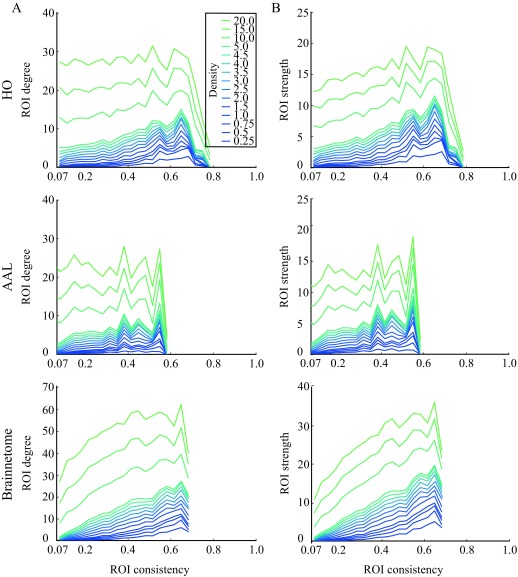
Degree and strength of ROIs increase with consistency. For degree (A), this behavior is mostly visible only at low and intermediate network densities, whereas strength (B) and consistency correlate even for high densities. Averaging and binning as in Figure 4.

The typical way of obtaining functional brain networks from correlation matrices is to threshold them so that only the strongest links are retained. We next threshold the ROI-level networks, in order to investigate whether the consistency of an ROI affects its network properties. For the thresholded networks, we calculate the mean degree (*k*) and strength (*s*) of ROIs across subjects. We observe that both the degree and the strength (Figure 7) increase with increasing consistency in networks thresholded to low and intermediate densities. For example, at *d* = 0.25%, the Pearson correlation coefficient for degree is in HO *r* = 0.39, *p* ≪ 10^−5^, in AAL *r* = 0.27, *p* ≪ 10^−5^, and in Brainnetome *r* = 0.53, *p* ≪ 10^−5^, and the Pearson correlation coefficient for strength is in HO *r* = 0.39, *p* ≪ 10^−5^, in AAL *r* = 0.28, *p* ≪ 10^−5^, and in Brainnetome *r* = 0.53, *p* ≪ 10^−5^.

Above, we showed that ROI-level correlations between pairs of ROIs appear rather independent of their consistencies. This may appear at odds with the degree and strength increasing with consistency; however, the independence of ROI-level correlations on consistency shows in full correlation matrices. When the correlation matrices are thresholded to retain only the strongest links, it becomes evident that these strongest links often connect to high-consistency ROIs.

In networks thresholded to higher densities, degree and strength are more independent of consistency. In HO and AAL, at the network density of *d* = 20%, no correlation is present between neither ROI consistency and degree (HO: Pearson correlation coefficient *r* = −0.055, *p* = 0.81; AAL: *r* = −0.039, *p* = 0.13) nor ROI consistency and strength (HO: *r* = 0.098, *p* = 3.2 × 10^−5^; AAL: *r* = 0.049, *p* = 0.057). In Brainnetome, a weak correlation between consistency and degree remains at density of *d* = 20 (*r* = 0.23, *p* ≪ 10^−5^), and consistency and strength are correlated both at density *d* = 20 (*r* = 0.036, *p* ≪ 10^−5^) and in the full network (*r* = 0.19, *p* ≪ 10^−5^).

## DISCUSSION

### Variation in Consistency of ROIs Points Towards Differences in Their Functional Homogeneity

The common approach of averaging over voxel time series in order to obtain representative time series for entire ROIs does not account for possible variations and inhomogeneities within the ROI. Therefore, to measure the level of such inhomogeneities, we introduced the concept of ROI consistency (*ϕ*) that is defined as the mean Pearson correlation coefficient between the time series of voxels in the ROI. This measure is simple to calculate and easy to interpret. If an ROI has high consistency, one can assume that the averaged time series accurately represent the underlying voxel dynamics. On the other hand, low consistency can be seen as indicative of functional inhomogeneity in the ROI.

Earlier, measures similar to ROI consistency have been used to quantify multiple aspects of the human brain function. For example, Li et al. (2002) have suggested that the consistency of the hippocampus could be used as an early-stage biomarker for Alzheimer’s disease.

We investigated ROI consistency in three different atlases: the anatomical HO and AAL atlases and Brainnetome atlas that is based on structural and functional connectivity. We found that distribution of consistencies across ROIs is broad in all these atlases. The most consistent ROIs are twice as consistent as the least consistent ones. This is in line with earlier observations (Baumgartner, Somorjai, Summers, & Richter, 1999; Craddock et al., 2012): Voxel time series within an activated brain area are not always strongly correlated. Further, the anatomical distribution of consistency is not uniform. In contrast to what one might expect, we found no significant difference between the Brainnetome atlas and the anatomical atlases in terms of mean consistency of ROIs.

### What Does Consistency Say About the Properties and Functional Role of an ROI?

It is tempting to interpret the consistency as a measure of the *goodness* of an ROI: ROIs with high consistency are well-defined, whereas low consistency indicates inaccuracies in the ROI definition. Indeed, a measure similar to mean consistency across ROIs has been used to quantify the overall accuracy of a parcellation (Craddock et al., 2012; Stanley et al., 2013; Thirion et al., 2006; see also Gordon et al., 2014 for a different definition of ROI homogeneity based on network connectivity). However, this interpretation, although useful, is probably oversimplified.

The nonuniform anatomical distribution of consistency opens the question of its dependency on time and task. Does the consistency of an ROI vary in time and how is it determined at a given moment? One plausible hypothesis is that active brain areas have higher consistency than currently inactive areas. This would also explain the dependency between consistency and degree of ROI at low-to-intermediate network densities. More active ROIs that should have higher consistency also have stronger momentary functional connections to other active ROIs and thus have a higher degree.

The concept of regional homogeneity (ReHo) (Zang, Jiang, Lu, He, & Tian, 2004; see also Jiang & Zuo, 2016 for a review on how ReHo has been used) is based on an assumption similar to the above hypothesis on the increased consistency of active brain regions. In the ReHo framework, active voxels are identified by their increased similarity to their neighbors. This similarity is calculated in a cubic neighborhood that includes the voxel itself and its neighbors in terms of faces (7 voxels); faces and edges (19 voxels); or faces, edges, and corners (27 voxels). Despite minor technical differences—in ReHo, Kendall’s coefficient of concordance is used to quantify the similarity of voxel time series—the similarity of ReHo and ROI consistency is obvious: If we define an ROI that covers the ReHo neighborhood of a voxel, the consistency of this ROI is proportional to the ReHo value of the centroid voxel.

ReHo has been used to quantify the changes in local connectivity not only in single voxels but also in brain areas. Jiang et al. (2015) calculated ReHo using a two-dimensional reconstruction of the cortex, and averaged the ReHo values across voxels within functionally defined subareas in the ventral visual system, the prefrontal cortex, and the posteromedial cortex. The differences of the mean ReHo between the subareas, obtained during resting state, reflected the different roles of these subareas in hierarchical information processing.

Despite the obvious similarities between consistency and ReHo, the purpose of these two measures is different. ReHo is a voxel-level measure that is always calculated across the immediate neighborhood of a voxel. Originally, ReHo was designed to localize activity to a handful of voxels that may be spatially separated (Zang et al., 2004), and it can be used also to construct functional brain networks at the level of voxels (Jiang et al., 2015). In contrast, consistency is a property of a collection of voxels (ROI) and can be used to quantify how well the average signal of these voxels represent the original voxel-level dynamics. Therefore, the usage of consistency is by definition limited to cases where activated brain areas—and nodes of functional brain networks—are assumed to be larger than single voxels. As consistency is by definition an ROI-level measure, it is natural that it relates to properties of ROI-level brain networks, for example, node centrality measures and strength of links.

Bearing these differences between ReHo and consistency in mind, it is logical that an ROI with low consistency can still contain voxels that have high ReHo; this is the case if the ROI definition is not accurate and the ROI contains subareas of internally correlated voxels. If the boundary between these subareas is sharp enough, even the mean ReHo of this low-consistency ROI can be high. Similarly, an ROI with high consistency can contain low-ReHo voxels: One can expect to find these at least at the boundaries of the ROI where the ReHo neighborhood contains voxels from different ROIs.

Task-induced changes in the consistency of an ROI could also reveal its functional role, similar to the mean ReHo in the study of Jiang et al. (2015). On one hand, if an ROI consists of functionally different subareas, some tasks may require synchronous activation of them all, yielding high consistency. On the other hand, in some conditions these subareas may activate at different times, which results in lower consistency. Therefore, the application of the concept of consistency beyond single ROIs, such as to resting state or task-related networks,may reveal dynamics of coactivation and separation of different subnetworks.

High values of test-retest reliability have been reported for several measures of local connectivity, in particular for ReHo (Zuo & Xing, 2014; Zuo et al., 2013). In the present study, we do not investigate the test-retest reliability of consistency: When consistency is used to evaluate the level of within-ROI homogeneity, low consistency values measured in one resting-state session can be viewed as a sufficient counterexample to the assumption of homogeneous voxel dynamics within an ROI. However, when task-dependency of consistency is investigated and, in particular, if one assumes that high consistency is indicative of increased activity, it is important to address the question of the test-retest reliability of the measure. In other words, before investigating the changes of consistency between rest and task, it might be good to ensure that consistency is stable between different resting-state sessions of the same subject. This investigation should also address the possible effects of preprocessing on the test-retest reliability, as Zuo et al. (2013) have done for ReHo.

### The Consistency of ROIs Is Affected by Their Shape and Size and the Level of Spatial Smoothing

There are multiple factors that can explain the consistency of an ROI. First, even if one assumes that perfectly consistent and static ROIs exist, atlases have typically been constructed at group level and do not perfectly match any individuals. Because of this, an ROI may overlap with many true functional subareas that are internally coherent, and following this, small ROIs are less likely to consist of several subareas. Similar results have been obtained by Gordon et al. (2014) using a network-level homogeneity measure. Furthermore, adjacent voxels are known to be more strongly correlated (Alexander-Bloch et al., 2013; Salvador et al., 2005). Therefore, small ROIs should be more consistent. Additionally, the shape of an ROI may affect its consistency as well: Small, spherical ROIs should be most consistent.

Interestingly, we found that consistency depends on ROI size in the anatomical atlases but not in Brainnetome. In Brainnetome, ROIs are on average smaller than in HO and AAL and the variation of ROI size is also smaller, which may explain this difference between atlases (Gordon et al., 2014). However, it is also possible that in the connectivity-based Brainnetome atlas, ROI consistency is less explained by size and more by other features such as the functionality of the ROI.

Spatial smoothing is commonly used in the preprocessing of fMRI data. We found that spatial smoothing increases the consistency of practically every ROI. However, smoothing does not make the consistency distribution significantly more narrow. Even when smoothing is applied, the consistency values of the least consistent ROIs are only half of those of the most consistent ROIs.

From the viewpoint of voxel signals, the effects of spatial smoothing are qualitatively similar to those of averaging voxel signals in order to obtain ROI time series: Any shared signal components are amplified, whereas individual components are suppressed. Therefore, spatial smoothing increases the correlations between all voxel time series. This increase is strongest for voxels that are less than a full width at half maximum (FWHM) apart, which is typically the case for voxels within the same ROI. In conclusion, one may argue that spatial smoothing is redundant when constructing ROI-level networks: Noise suppression is already taken care of when the voxel-level signals are averaged to ROI-level signals, and smoothing basically only results in a baseline shift in ROI consistencies.

### Averaging of Voxel Signals May Produce Spuriously High ROI-Level Correlations

We demonstrated that there are pairs of low-consistency ROIs that have strong ROI-level correlations; in general, ROI-level correlations are scattered and do not appear to depend on ROI consistency. By contrast, the mean consistency of an ROI pair determines an upper limit for their voxel-level correlation. The existence of spuriously high ROI-level correlations is explained by the amplification of shared voxel-level signal components when the voxel signals are averaged in order to obtain the ROI time series. At the same time, averaging suppresses individual components.

Averaging related to moving from the voxel level to the ROI level can be seen from two different angles. First, if we assume that ROIs represent functionally uniform groups of voxels that have an underlying common signal component, then the individual voxel-level signal components can be considered as noise. In this case, averaging acts as a filter that suppresses noisy signal components and increases the signal-to-noise ratio (SNR). On the other hand, individual voxel-level signal components can also reflect true underlying neural activity, and ROI-wide shared components can be caused by physiological noise or external noise. Should this be the case, averaging of the voxel signals would cause a loss of information. However, proving any of the above points of view would require more detailed modeling of changes in SNR when voxel signals are averaged to obtain the ROI time series.

As moving from the voxel level to the ROI level changes the link structure of the network, one may expect to see differences in other network properties as well. Indeed, Hayasaka & Laurienti (2010) have demonstrated that voxel and ROI-level functional brain networks differ from each other in terms of several network metrics such as degree distribution, characteristic path length, and local and global efficiency. These metrics naturally depend on the link structure of the network and may therefore have been affected by the structural changes caused by averaging voxel signals. Based on their results, Hayasaka & Laurienti (2010) decided to recommend against using anatomical ROIs as nodes of functional brain networks.

The voxels used in the acquisition and analysis of fMRI data are artificially defined, and it is reasonable to assume that the true functional areas in the brain are larger than voxels (Shen et al., 2013; Wig et al., 2011). Therefore, coarse-graining of voxels into well-defined larger-scale regions can yield more accurate results than voxel-level analyses. However, we have shown that all ROIs are not equally consistent. Therefore, when working with ROIs, one should be careful in interpreting results and aware of possible problems, such as spuriously high correlations between inconsistent ROIs.

## MATERIALS AND METHODS

### Subjects

Thirteen healthy, right-handed subjects (11 females, 2 males, age 25.1 ± 3.9, mean ±*SD*) participated in the study of emotion processing. All subjects had normal or corrected-to-normal vision, and none of them reported a history of neurological or psychiatric disease. All subjects were volunteers, gave written, informed consent according to the Declaration of Helsinki, and were compensated for their participation. The study was approved by the Research Ethics Committee of Aalto University.

### Data Acquisition

Functional magnetic resonance imaging (fMRI) data were acquired with a 3T Siemens Magnetom Skyra scanner in the AMI Centre (Aalto Neuroimaging, Aalto University, Espoo, Finland). A whole-brain T2*-weighted EPI sequence was measured with the following parameters: TR = 1.7s, 33 axial slices, TE = 24 ms, flip angle = 70°, voxel size = 3.1 × 3.1 × 4.0 mm^3^, matrix size 64 × 64 × 33, FOV = 256 × 256 mm^2^. Data from an approximately 6 min (215 time points) resting-state session were used in this study. During the resting state, subjects were asked to lay still with their eyes open, fixating to a gray background image, and avoid falling asleep.

Besides fMRI, anatomical MR images with isotropic 1 × 1 × 1 mm^3^ voxel size were also acquired using a T1-weighted MP-RAGE sequence.

### Preprocessing of the Data

The fMRI data were preprocessed with FSL (Jenkinson, Beckmann, Behrens, Woolrich, & Smith, 2012; Smith et al., 2004; Woolrich et al., 2004) and with an in-house MatLab toolbox, BraMiLa (https://version.aalto.fi/gitlab/BML/bramila). The preprocessing pipeline began with removal of the three first frames of each subject’s data in order to eliminate the error caused by scanner transient effect (leaving 212 time points for further analysis), slice timing correction, motion correction by MCFLIRT (Jenkinson, Beckmann, Behrens, Woolrich, & Smith, 2002), and extraction of white matter and cerebrospinal fluid (CSF). Functional data were coregistered to the anatomical image with FLIRT (7 df), further registered to MNI152 2 mm standard template (12 df), and resampled to voxels of 4 × 4 × 4 mm^3^. Signals were linearly detrended, and signals from white matter and CSF were regressed out from the data.

Regressing out from the data, the global signal (GS) decreases motion-related variance of the data (Power et al., 2014). However, removal of GS may also distort correlation patterns in the network (Fox, Zhang, Snyder, & Raichle, 2009; Gotts et al., 2013). Therefore, there is no general consensus among the fMRI community whether GS should be regressed out. In the present work, we decided not to regress out GS. The potential effect of GS on our data would be increased ROI consistency (see below), since GS is shared among all voxels and therefore may increase voxel-level correlations. However, we observed a wide range of ROI consistency values, including very low consistencies.

In order to control for motion artifacts, expansion of motion parameters was extracted out from the data with linear regression (36 Volterra expansion-based signals; Power et al., 2014). Head motion has been reported as a source of artifacts in connectivity studies (Power, Barnes, Snyder, Schlaggar, & Petersen, 2012). Therefore, we calculated the framewise displacement for each subject. However, as the framewise displacement of all the subjects was under the suggested threshold of 0.5 mm, we did not perform any scrubbing. Further, in later analysis we also investigated whether differences in head motion could explain differences in ROI consistency.

In order to further avoid artifacts, voxels that were located at the edge between brain and skull where the signal power was less than 2% of the individual subject’s mean signal power were excluded from further analysis.

### Spatial Smoothing

Our standard preprocessing pipeline did not include spatial smoothing. However, in order to investigate how this commonly used preprocessing method would affect our results, we repeated all analysis with data that had been smoothed with a Gaussian kernel. We used three different kernel sizes: full width at half maximum (FWHM) of 5 mm, 8 mm, and 12 mm. For details, see Supplementary Methods (Korhonen et al., 2017).

### Atlas-Based Regions of Interest

After preprocessing (and spatial smoothing when applied), the cerebral cortex as well as subcortical structures and the cerebellum were divided into ROIs. For defining the ROIs, we used three different brain parcellations: the commonly used anatomical HO and AAL atlases as well as the Brainnetome atlas that is based on structural and functional connectivity.

The ROIs were defined so that each voxel belonged to one ROI only. The time series of ROIs were defined as averages of the time series of the voxels within the ROIs:

$$X_{I}=\frac{1}{N_{I}}\underset{i\in I}{\sum }x_{i},$$ (1)

where *I* is the focal ROI, *N*_*I*_ is its size defined as the number of constituent voxels, and *x*_*i*_ is the time series of voxel *i*.

#### Harvard-Oxford atlas

HO atlas (http://neuro.debian.net/pkgs/fsl-harvard-oxford-atlases.html; Desikan et al., 2006) is a probabilistic atlas that is based on the macroanatomical boundaries of the brain. In the present study, we used 138 ROIs from the HO atlas at a probability level of 30% (meaning that each voxel belongs to the ROI it has been associated with in 30% or more of the subjects in the group used to create the parcellation). Ninety-six of these ROIs were located at the cerebral cortex, 15 of them covered subcortical gray matter, and 27 were located in the cerebellum. Note that one of the cerebellar ROIs of the HO atlas (ROI 120, Vermis Crus I) is not defined at the probability level of 30% and was therefore excluded from the present study.

In the HO atlas, the distribution of ROI sizes is broad: in the case of our in-house data, the number of voxels in an ROI varied between 5 and 857, median ROI size being 88 (for sizes of all ROIs, see Supplementary Table; Korhonen et al., 2017).

#### Automated Anatomical Labeling atlas

ROIs of the AAL (Tzourio-Mazoyer et al., 2002) atlas have been obtained by parcellating a spatially normalized high-resolution single-subject structural volume based on the main sulci. Then, a label has been automatically assigned to each of the ROIs. In the present study, we used 116 ROIs from the AAL parcellation. Ninety of these ROIs were located at the cerebral cortex, while 8 of them consisted of subcortical gray matter, and 18 covered the cerebellar cortex.

As in the HO atlas, in AAL the size of ROIs varied across a wide range. In the case of our in-house data, the number of voxels in an ROI varied between 4 and 607, with a median of 157 (for further details, see Supplementary Table; Korhonen et al., 2017).

#### Brainnetome atlas

The Brainnetome atlas (Fan et al., 2016) is based on in vivo structural and functional connectivity measured using multimodal neuroimaging techniques. In the present study, we used 246 ROIs from the Brainnetome atlas, 210 of which were located at the cerebral cortex and 36 covered subcortical gray matter. Note that the Brainnetome atlas does not include ROIs located in the cerebellum.

In Brainnetome, the size of ROIs varied less than in HO or AAL: In the case of our in-house data, the minimum number of voxels in an ROI was 5 and the maximum number of voxels in an ROI was 186, with a median of 63. So, Brainnetome ROIs were smaller than ROIs of HO or AAL.

### Network Extraction

We study functional brain networks at two different scales: at the level of voxels and at the level of ROIs. At the voxel level, nodes of the network represent single voxels of the cortical gray matter, whereas at the ROI level whole ROIs are used as network nodes. At both levels, network extraction begins with calculating the weighted adjacency matrix *A* between all nodes so that its element *A*_*i*,*j*_ indicates the strength of connection between nodes *i* and *j* of the network. To define the connection strength, we calculate the Pearson correlation coefficients between time series of each *i*-*j* pair. Pearson correlation is commonly used to quantify functional connections between nodes of brain networks (see Braun et al., 2012; Wig et al., 2011). This procedure yields a full, symmetric, weighted adjacency matrix that, after removing the diagonal values, contains $\frac{1}{2}N(N-1)$ independent real-valued elements where *N* denotes the number of network nodes.

We performed most of our analyses on the full adjacency matrix, using zero-lag correlations between all nodes of the network. However, for the analysis of the relationship between consistency, degree, and strength of an ROI (see below), the network was thresholded to a set of densities (0.25, 0.5, 0.75, 1, 1.5, 2, 2.5, 3, 3.5, 4, 4.5, 5, 10, 15, and 20%). To obtain a network with a density *d*, links weaker than the 1 − *d*th percentile were removed from the network by setting the corresponding elements in *A* to zero. Thresholded networks were analyzed as weighted, instead of transforming the thresholded adjacency matrix into a binary one. The range of densities was chosen to emphasize low densities, where network structure is most sensitive to small changes in link weights.

The neuroscientific interpretation of negative correlations between brain areas has been subject to dispute. It has been argued that negative correlations are less reliable than positive ones and should thus be excluded from analysis (Schwarz & McGonigle, 2011; Shehzad et al., 2009). At the same time, others have argued that negative correlations have a true neurobiological meaning (Fox et al., 2009; Wig et al., 2011). Negative correlations are indeed included in our analysis of full matrices, that is, calculations of distributions of correlations between time series of voxels in same and different ROIs, and studies of the relationship between consistency and voxel and ROI-level correlations. In contrast, for thresholded networks that are used to investigate the relationship between consistency and network measures, the thresholding method automatically excludes negative correlations since only the strongest *d*% of links are accepted at each density *d*.

### The Consistency of ROIs

In order to quantify the amount of variation between the time series of the voxels within an ROI, we define the ROI consistency as

$$\phi (I)=\frac{1}{N_{I}(N_{I}-1)}\underset{i,i\prime \in I}{\sum }C(x_{i},x_{i\prime }),$$ (2)

where *I* denotes the focal ROI, *N*_*I*_ is the number of voxels in *I*, *C*(*x*_*i*_,*x*_*i*′_) denotes the Pearson correlation coefficient between the time series *x*_*i*_ and *x*_*i*′_ of voxels *i* and *i*′ that belong to *I*, and the summation is done over all voxel pairs within *I*. The interpretation of consistency is straightforward: If an ROI contains many voxels with nonuniform dynamics, *ϕ* is low, indicating that the ROI time series is a poor approximation of voxel dynamics. In contrast, an ideal ROI where all voxels have identical time series would reach the theoretical maximum value of *ϕ* = 1.

Negative values of consistency are possible only in theory. They arise only when the ROI consists of pairs of voxels with signals anticorrelated with each other and independent of the signals of the rest of voxels in the ROI. In practice, there are only a few significant negative correlations between signals of voxels within an ROI (see Figure 1), and we did not observe any negative consistency values.

### Time Series Correlations at the Voxel and ROI Levels

In the present article, we ask whether consistency predicts the connectivity of an ROI. We do this by investigating the relationship between the mean consistency and correlation between an ROI pair at two levels. The *voxel-level correlation* between two ROIs is defined as the mean correlation between the time series of voxels within the ROIs, averaged over all possible voxel pairs:

$${\langle C\left(I,J\right)\rangle }_{vox}=\frac{1}{N_{I}N_{J}}\underset{i\in I}{\sum }\underset{j\in J}{\sum }C(x_{i},x_{j}).$$ (3)

The *ROI-level correlation* between two ROIs is simply defined as the Pearson correlation between the time series of the ROIs:

$$C{\left(I,J\right)}_{ROI}=C(X_{I},X_{J}),$$ (4)

where *X*_*I*_ and *X*_*J*_ are the ROI time series, computed as averages of voxel time series within *I* and *J* (see Equation 1).

### Relationship Between ROI Consistency and Network Measures

In order to investigate the role of an ROI as a network node, we utilized two commonly used network measures: the degree *k* and the strength *s* of an ROI. These measures are commonly used to identify the hubs of the functional brain network (Rubinov & Sporns, 2010).

The degree of an ROI is defined as the number of its neighbors, that is, the number of other ROIs to which it is directly connected by a link. The degree offers a rough estimate of how central a node is in the network: Nodes with many neighbors can be seen as more important for information flow than nodes with less neighbors.

The strength of an ROI is defined as the sum of the weights of the links of the ROI. Thus, it quantifies the amount of connectivity of the ROI. Like the degree, the strength of an ROI can be used to estimate its centrality; unlike the degree, it accounts for link weights too. However, because strength mixes topology (existence of links) with link weights, interpretation is not always straightforward: An ROI with a few strong links can have the same strength as an ROI with many weaker links.

As discussed above, high ROI consistency is expected to be related to strong voxel-level correlations. It is also natural to assume that strong voxel-level correlations give rise to strong ROI-level correlations. Thereby, a natural hypothesis would be that ROIs that have high consistency are connected by strong links. As a result, these ROIs can be expected to have high strength, too. In a thresholded network, higher strength may also indicate larger number of links with above-threshold weight, giving rise to higher degree as well. We have investigated these relationships in networks thresholded to different densities.

### ABIDE Data

In order to ensure that our results are not caused by any property specific to our in-house dataset, we repeated all our analysis for a second, independent dataset. This dataset, referred to as the ABIDE data, was part of the Autism Brain Imaging Data Exchange I (ABIDE I) project (Di Martino et al., 2014) and consisted of 28 healthy controls. For details of the ABIDE data, see Supplementary Methods (Korhonen et al., 2017).

## ACKNOWLEDGMENTS

We acknowledge the computational resources provided by the Aalto Science-IT project. We thank Rainer Kujala for inspiring discussions and for comments on the manuscript, and Marita Kattelus and Athanasios Gotsopoulos for their help in data acquisition.

## AUTHOR CONTRIBUTIONS

Onerva Korhonen: Conceptualization: Lead; Formal analysis: Lead; Investigation: Lead; Meth odology: Lead; Software: Lead; Visualization: Lead; Writing – original draft: Lead; Writing – review & editing: Lead. Heini Saarimäki: Data curation: Lead; Resources: Lead; Writing – review & editing: Supporting. Enrico Glerean: Software: Equal; Writing – review & editing: Supporting. Mikko Sams: Conceptualization: Equal; Supervision: Lead; Writing – review & editing: Supporting. Jari Saramäki: Conceptualization: Lead; Supervision: Lead; Writing – original draft: Supporting; Writing – review & editing: Lead.

## TECHNICAL TERMS

Spurious link: A connection that is not in reality present in the underlying data but appears in the extracted network.
Functional homogeneity: Every voxel of an ROI is performing some particular function and therefore has (roughly) similar dynamics, yielding strongly correlated voxel time series.
Thresholding: Discarding weak links in order to make the backbone of a network more visible.
Hubs: Most central nodes of a network, via which most of the information flowing in the network is transmitted.
Degree: The number of neighbors a node has.
Strength: The sum of the weights of the links attached to a node.
